# Egg White-Based Gels with Candelilla Wax: A Study of Rheological, Mechanical, Calorimetric and Microstructural Properties

**DOI:** 10.3390/gels10110733

**Published:** 2024-11-13

**Authors:** Iram Cisneros-García, Ma. de la Paz Salgado-Cruz, Alitzel B. García-Hernández, Gustavo F. Gutiérrez-López, Humberto Hernández-Sánchez, Brenda H. Camacho-Díaz, Liliana Alamilla-Beltrán

**Affiliations:** 1 Departamento de Ingeniería Bioquímica, Escuela Nacional de Ciencias Biológicas, Instituto Politécnico Nacional, Av. Wilfrido Massieu 399, Gustavo A. Madero, Ciudad de Mexico 07738, Mexico; icisnerosg1500@alumno.ipn.mx (I.C.-G.); ggutierrezl@ipn.mx (G.F.G.-L.); hhernadezs@ipn.mx (H.H.-S.); 2 CONAHCyT-Centro de Investigación en Química Aplicada (CIQA), Parque de Investigación e Innovación Tecnológica, Av. Alianza Sur 204, Apodaca 66628, Mexico; ali_ialee@outlook.com; 3 Centro de Desarrollo de Productos Bióticos, Instituto Politécnico Nacional (IPN), Carretera Yautepec-Jojutla Km. 6, Calle CEPROBI No. 8, San Isidro, Yautepec 62731, Mexico; bcamacho@ipn.mx

**Keywords:** bigels, oleogel, hydrogel, texture

## Abstract

Bigels (BGs) are innovative composite systems that integrate oleogel and hydrogel structures, and are gaining increasing attention for their unique textural and functional properties in food applications. This study evaluated the rheological and mechanical properties of egg white-based bigels incorporating candelilla wax (CW) as an oleogelator. The results indicate that different egg white protein (EWP) (5–10%) concentrations and hydrogel-to-oleogel ratios (20:80 to 80:20) significantly influenced the structural and functional properties of the bigels. Compression testing revealed no significant differences in strength across the tested range; however, higher EWP concentrations enhanced the stability of the BGs. Furthermore, increased candelilla wax oleogel (CWO) content (60%) markedly improved emulsion stability, resulting in superior strength, as confirmed by dynamic light scattering. Rheological studies demonstrated shear-thinning behavior, particularly at higher hydrogel content related to the oleogel (W/O), which exhibited the highest yield stress. Microstructural investigations confirmed the presence of a continuous oleogel phase within the bigels (W/O) and revealed the formation of a complex structure. These findings suggest that a reduced hydrogel-to-oleogel ratio can be utilized across various food systems, opening new possibilities for creating customized food structures with desirable textural and functional attributes.

## 1. Introduction

Public health problems, including cardiovascular diseases, obesity and elevated LDL cholesterol levels, have been increasingly associated with the consumption of foods rich in saturated and trans fatty acids [1,2]. In response, the field of food science is actively pursuing alternatives and strategies to mitigate the use of these fats. However, elimination of saturated and trans fats poses challenges due to their considerable advantages; unlike oils, these fats enhance structural properties (e.g., consistency, hardness, brittleness) and sensory attributes (e.g., taste, appearance, odor) of food products. 

Recent studies have investigated methods to modify the physical properties of oils, particularly through hydrogenation. This process increases the saturation of carbon–carbon double bonds and converts *cis*-geometric isomers into more stable *trans*-isomers, although with alterations in the chemical structure of the oils. Consequently, the search for safe alternatives to *trans* fats continues. A promising approach involves the utilization of gel systems characterized by firmness and resilience, which may serve as effective substitutes for conventional fats [3].

Gels consist of three-dimensional networks of flexible chains swollen by a liquid. The composition of the continuous phase distinguishes various gel systems. This phase comprises a network of polymers or gelators that entrap water or oil, influencing the gel’s texture and functionality. For instance, hydrogels are three-dimensional hydrophilic networks capable of absorbing significant amounts of water without dissolving [4]. In contrast, oleogels are semi-solid, hydrophobic materials that retain a high proportion (≥90%) of edible liquid oil within a three-dimensional structure formed by oleogelators, such as waxes [5]. These oleogels improve oxidative stability, firmness and spreadability, making them suitable substitutes for saturated fats [6]. Candelilla wax (CW) has been recognized as an effective oleogelator due to its high content of n-alkanes, triterpenes, triterpenic alcohols and esters [7,8]. Its multi-component structure and medium melting point allow stable gel formation at low concentrations (<1 w%) [9], making it a suitable fat substitute in food, improving texture and reducing fat content [10,11,12]. In addition, CW interactions with polysaccharides [13] and proteins [9] significantly modify the oleogel microstructure, affecting thixotropy and stability [14]. These allow the creation of novel gels with tailored properties, which could also be used in pharmaceutical applications.

Recently developed composite systems, known as bigels, integrate oil structuring (oleogelation) and hydrogel formation, providing versatile rheological properties that favor the development of novel food products, particularly in bakery applications. BGs can be classified into oleogel-in-hydrogel, hydrogel-in-oleogel, or bicontinuous structures [15]. These systems address consumer demand for low-fat, nutritionally rich foods that retain desirable textures [16].

Typical gelling agents in hydrogel production include gelatin, guar gum, starch, κ-carrageenan, collagen, alginate, and whey protein isolate [4,17,18,19,20,21]. Additionally, EWPs are emerging as a promising alternative due to their exceptional gelling, emulsifying and foaming properties, which facilitate the formation of structural matrices capable of retaining water and essential nutrients [22]. EWPs typically form gels through heat-induced gelation [23], making them suitable for hydrogel formation in BGs. However, the effects of combining hydrogel and oleogel using EWPs on the mechanical properties of BGs have not yet been thoroughly investigated, presenting a novel opportunity to take advantage of the unique characteristics of EWPs in BGs formulations.

In this regard, this study aimed to examine the interactions between EWPs and CW concerning the physical, structural, textural, rheological, and mechanical properties of BGs. For this purpose, low-cost, reproducible, stable and suitable saturated fat alternatives were developed for incorporation into food products, systems that could be classified as Generally Recognized As Safe (GRAS). Finally, for food-grade BGs to be effectively used as functional food systems and accurately reflect the dynamic environments encountered in industrial applications, it is essential to understand their formation processes, including rheological, structural, and thermal conditions. Therefore, this work addresses a comprehensive understanding of these factors, optimizing and demonstrating their performance in real-world applications.

## 2. Results and Discussion

### 2.1. Compression Testing of Bigels

**Figure 1:**
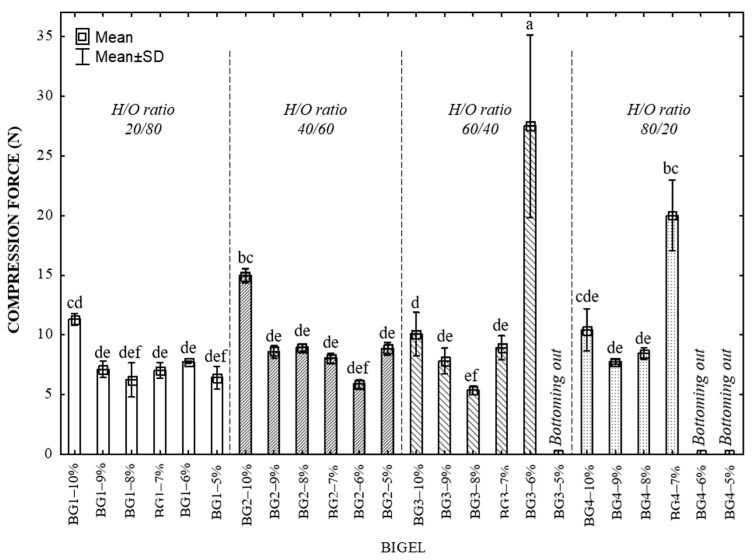
Comparison of compression force in bigels at different protein concentrations and H/O ratios. Means with different letters differ significantly (*p* < 0.05, Tukey’s test). SD, Standard Deviation.

Figure 1 illustrates the effects of protein concentration and oleogel ratio on the textural properties and structural integrity of EWP-based BGs. The maximum compression force exerted during the initial compression cycle is a crucial parameter [24]. Notably, no statistically significant differences in firmness (*p* < 0.05) were observed among BGs with varying EWP concentrations and hydrogel/oleogel (H/O) ratios. However, a slight reduction in firmness correlated with lower protein concentrations, likely due to decreased protein aggregation and post-denaturation hydrophobic interactions, resulting in a less compact structure, a lower void density (due to air incorporation during emulsification), and a less stable gel capable of retaining water and oleogel, consistent with findings by Cui et al. [25].

A bottoming-out effect and biased measurements were observed in some results due to the lower protein and oleogel concentrations and higher water content. These conditions require substantial compression forces to break the gel, leading to instability. This behavior was evident in the BG sample with 6% and 7% protein (BG3–6% and BG4–7%), which showed large standard deviations. Similar tendencies were observed for BG3–5%, BG4–6% and BG4–5%, where higher hydrogel ratios (60:40 and 80:20 H/O) combined with low EWP led to weakened structures.

**Figure 2:**
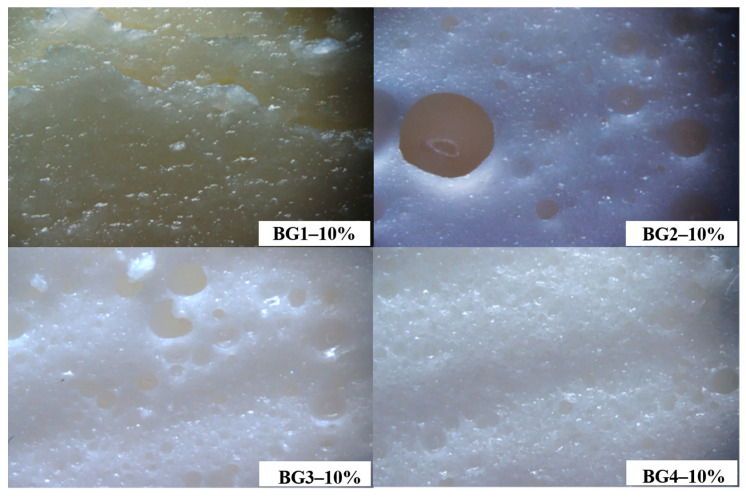
Images of the surface structure of the bigels were captured using a stereoscope.

Upon emulsification, the BG composed of 20% hydrogel and 80% oleogel, displayed phase separation. This phase separation occurs during heat treatment, which triggers the release of loosely bound wax within the protein network. Statistical analysis of BG1–10%, BG2–10%, BG3–10%, and BG4–10% bigels revealed significant differences due to the increased hydrogel phase (H-phase), yielding final protein concentrations of 2.18%, 4.36%, 6.55%, and 8.72%, respectively (Table 1). Higher protein content produced a densely packed network with numerous small voids or pores (Figure 2). The formation and stabilization of the foam are likely attributed to ovomucins, the EWPs that readily lose their globular structure during heat treatment [26].

The concentration of EWPs is crucial for the gelation and structural integrity of BGs. Higher protein levels promote the formation of a dense network, enhancing firmness, compressive strength, and hydrogen and hydrophobic bonding. This effect is further supported by the content of CW in the oleogel phase, which improves mechanical stability by interacting with EWPs to create a stable network during emulsification and thermal treatment, as evidenced by the increased compressive strength in samples BG1–10% and BG2–10%. In this context, a higher oleogel content enhances extensibility [27], while greater hydrogel content improves resistance. Firmness, which correlates closely with the hardness test, affects the spreadability of fatty products such as bigels [28]. Butters with firmness in the range of 4–12 N have optimal malleability and can be easily spread on bread and other surfaces without cracking, according to Barroso et al. [17]. It is worth noting that the firmness values of samples BG2–10%, BG3–10% and BG3–8% are comparable to those of butter, indicating a softer texture that improves the palatability of fatty foods. Consequently, these samples were selected as model systems for rheological, thermal, and structural evaluation, facilitating further characterization and optimization of gel properties for specific applications. These properties are essential for the R&D of new food products, ensuring processing efficiency and consumer acceptance [29].

### 2.2. Characterization of the Emulsions Forming the Basis of Bigels

The evaluation of contact angle, rheology, and stability of emulsions in model systems based on CWO and EWP was conducted to assess the basis of the BGs. The aim was to determine whether the contact angle is sufficient to generate effective emulsifying action, thereby reducing interfacial tension and stabilizing the emulsion. Rheological flow curves were studied to measure apparent viscosity and shear response, facilitating the optimization of processing and gelation parameters. Stability measurements ensured that CWO and EWP maintained emulsion uniformity during heat treatment. These results will help confirm that this type of BG system meets the desired functional and textural specifications for use in final products.

The contact angle measurements in Table 2 indicate that emulsions before heat treatment (BG2–10%, BG3–10%, and BG3–8%) exhibited contact angles below 90° with oil droplets, reflecting their lipophilic nature. In this context, emulsions are classified as hydrophilic if the contact angle (θ) with a water droplet is less than 90°, and hydrophobic if θ exceeds 90°. Similarly, they are deemed lipophilic if θ with an oil droplet is less than 90°, and hydrophilic if θ exceeds 90° [30].

The BG2–10% emulsion, containing 4.5% EWP, displayed water-in-oil (W/O or H/O) dispersion characteristics and exhibited a significantly higher contact angle compared to the oil-in-water (O/W or O/H) system BG3–8% (3% EWP). However, both emulsions remained hydrophilic, with contact angles below 90°. This behavior is attributed to the unfolding of protein structures, particularly lipophilic proteins, and the amphipathic properties of CW, which enhance the emulsion’s hydrophilic nature by interacting with proteins and water [7]. These results align with those reported by Dickinson and McClements [31,32], who found that lipophilic proteins particularly tend to orient themselves on the surface, effectively interacting with and partially enveloping the oil droplet. This orientation contributes to a smaller contact angle.

No significant difference in contact angle was observed between the BG2–10% and BG3–10% emulsions, indicating that increasing the aqueous phase (hydrogel) does not notably affect the lipophilicity of the emulsions. This stability can be attributed to the emulsifying properties of EWP and CW, which interact with the oil–water interface to stabilize the emulsion. However, a significant decrease in contact angle was observed when the protein concentration was reduced to BG3–8%, suggesting greater lipophilicity. This change results in a less effective interfacial barrier and increased exposure to the oil phase, which can impact the emulsion’s surface properties. Additionally, the amphiphilic nature of CW may further enhance the hydrophobicity of the emulsion [33]. 

All emulsions exhibited high hydrophilicity due to a stable interfacial film formed by the CW and EWP. This film exposed hydrophilic regions and resulted in contact angle values of less than one when evaluated with water droplets. This interfacial phenomenon optimizes hydrophilic interactions in W/O and O/W systems by aligning amphiphilic wax moieties with hydrophobic protein domains, resulting in a predominantly hydrophilic barrier that prevents oil phase interaction with water droplets.

Apparent viscosity analysis (Table 2) revealed significant differences between the (H/O) W/O BG2–10% emulsion and the O/W BG3–10% and BG3–8% emulsions, with the latter exhibiting low apparent viscosity. This behavior suggests that a higher oleogel fraction increases apparent viscosity, primarily due to the denser and more structured network provided by the oleogel phase, which resists flow and deformation. These findings align with those reported by Mata-Mota et al. [20]. Notably, no significant apparent viscosity difference was observed between BG3–10% and BG3–8%. Although previous studies [15,34] have reported that increasing hydrogelator concentrations generally lead to higher apparent viscosity, our results suggest that EWP concentration does not significantly influence apparent viscosity when the H/O ratio is constant. This effect may be attributed to the oleogel phase’s dominant role in determining structural characteristics and overall apparent viscosity.

**Figure 3:**
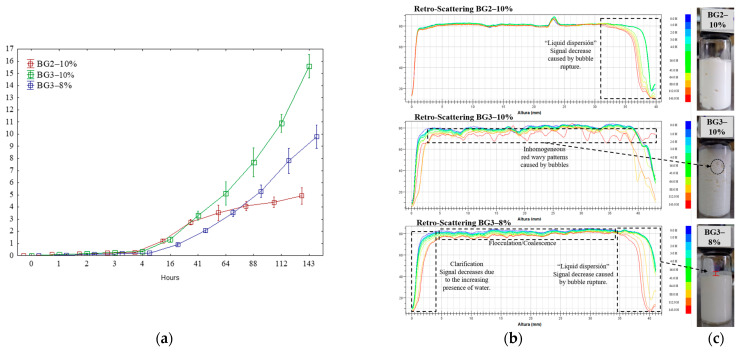
Turbiscan Stability Index (TSI) (**a**) and backscattering (BS) profiles (**b**) over 143 h of storage for emulsion formulations with varying hydrogel/oleogel ratios and EWP concentrations at 25 °C. Images of the emulsions after 143 h of storage (**c**).

Dynamic light-scattering measurements assessed the physical stability of the emulsions over a 143 h storage period, as depicted in Figure 3a. The Turbiscan Stability Index (TSI) revealed two stability phases for the three samples analyzed. The first phase exhibited TSI values > 1 for 16 h, while the second phase indicated values ranging from 1 to 15.58 ± 0.38 for the BG3–10% sample, suggesting increased instability [35]. The reduced stability of BG3–8% emulsions has been attributed to proteins with hydrophobic groups often concealed within their molecular structure, resulting in low surface hydrophobicity and poor emulsification [36]. Previous studies by Wijarnprecha et al. [37] also noted that proteins could hinder the emulsifying ability of certain oleogelators, such as waxes, leading to phase separation in the BG3–8% emulsion.

In contrast, BG2–10% (1 to 4.92 ± 0.27) exhibited superior stability, likely due to its higher oleogel content (60%), which enhances emulsion stability by creating a structured oil phase with increased apparent viscosity. This minimizes droplet movement, improves interfacial stability, and prevents creaming, aligning with findings by Alves-Barroso et al. [17]. The Turbiscan backscatter (BS) fingerprints for the BG2–10% emulsion (Figure 3b) corroborate the TSI results, indicating minimal destabilization through flocculation or coalescence, as evidenced by the consistent curves over time. This enhanced stability is likely attributable to the composition of CW and its concentration in the oleogel phase (O-phase). Key components such as fatty alcohols, wax esters, and free fatty acids interact with water and proteins in the emulsion, contributing to a structured matrix that stabilizes the oil–water interface. Binks and Rocher [38] demonstrated that wax, functioning as a hydrophobic particle, adsorbs at the oil–water interface, providing steric stability.

The drainage of foam (Figure 3c, red line), caused by either gravity or capillary forces, causes the thinning of the liquid films between air bubbles, leading to decreased stability (Figure 3b). This thinning results in two primary phenomena that contribute to foam collapse: (1) coalescence, where the thin films between neighboring bubbles break, forming more giant bubbles and reducing the volume of the foam; and (2) Ostwald ripening, which causes smaller bubbles to shrink while larger ones grow due to gas diffusion driven by pressure differences. Together, these processes lead to a reduction in the volume of the emulsion over time.

### 2.3. Thermal Characterization

**Figure 4:**
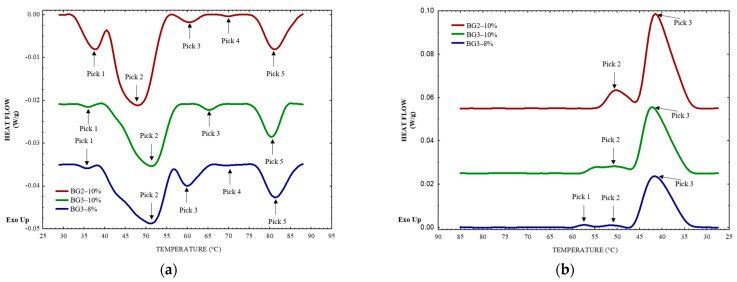
Thermograms of emulsion formulations with varying H/O ratios and EWP concentrations: (**a**) heating and (**b**) cooling phases.

Figure 4a,b show the thermal properties of the BGs. The initial three peaks observed during heating (Figure 4a) in the thermograms of all three systems are attributed to the content and melting of CW, with temperatures ranging from 35 to 65 °C. These findings are consistent with results from Rocha et al. and Serrato-Palacios et al. [39,40] for oleogels containing 4% *w*/*w* CW.

Notably, in Figure 4a, the temperature of the second peak for BG3–8% (52.36 °C) differs significantly from that of BG2–10% (48.13 °C). This shift can likely be attributed to the higher thermal conductivity of the aqueous phase components in BG3–8%, which contains 60% hydrogel. The thermal conductivity of water is approximately 0.63 W/m·K between 25 and 90 °C [41], while that of EWP is similar at around 0.59 W/m·K, decreasing to 0.5 W/m·K at 90 °C [42]. These components facilitate more efficient heat transfer, allowing CW in the O/W BG3–8% emulsion to melt at a lower temperature. In contrast, the W/O BG2–10% emulsion, in which the continuous phase is soybean oil with a thermal conductivity of about 0.156 W/m·K from 25 °C to 90 °C [43], requires higher temperatures for wax melting due to slower heat transfer. These highlight the critical role of phase composition and thermal properties in influencing wax melting behavior in emulsions. 

As reported by other authors, the melting profile of CW is mainly influenced by alkanes (C26–C33), especially hentriacontanes [40]. Meanwhile, the first endothermic peak (Figure 4a) corresponds to the transition of n-alkanes from crystalline (monoclinic) phases to an intermediate lamellar phase, also called a rotator phase [44]. The melting of this rotator phase is represented by the second peak. The third peak is associated with other components of CW, including wax esters, free fatty acids, long-chain alcohols, and triterpenoids. 

Denaturation of EWP is mainly responsible for peaks 4 and 5 in Figure 4b. The fourth peak at 71 °C is due to the denaturation of conalbumin. The fifth peak at 81 °C corresponds to the denaturation of ovalbumin, the main protein component of egg white. Typically, conalbumin and lysozyme show thermal denaturation at around 60–65 °C and 64–74 °C, respectively [45]. However, overlapping of the lysozyme transition by conalbumin complicates the precise observation of individual protein denaturation events. Ovalbumin denaturation is confirmed by the fifth peak [46]. These transitions support the formation of a protein network (EWP-based hydrogel).

The BGs exhibited no significant differences in cooling temperature peaks corresponding to the crystallization of CW (Figure 4b). The denatured EWP results in the formation of a gel network through irreversible hydrophobic interactions [47]. Therefore, no transitions concerning EWP were observed during cooling. In BG3–8%, an additional exothermic peak (peak 1) indicates that CW crystallization occurs at a higher temperature, attributed to the differing thermal conductivities of the oil and water phases. The oil droplets, surrounded by the aqueous phase, benefit from enhanced heat transfer and reduced thermal resistance, facilitating efficient thermal exchange, as mentioned above. Additionally, the interaction between the aqueous phase and wax plays a pivotal role in crystallization; research shows that proteins create a favorable environment for nucleation by providing sites that promote an orderly arrangement of wax molecules, leading to a more homogeneous crystalline structure [48,49,50,51]. Furthermore, the confinement of wax within the aqueous phase aids in sustaining a thermal gradient, which promotes effective energy dissipation and thereby enhances both nucleation and crystal growth. Finally, BGs (hybrid gels) formation is confirmed at this stage.

### 2.4. Rheological Properties

#### 2.4.1. Flow Curves

**Figure 5:**
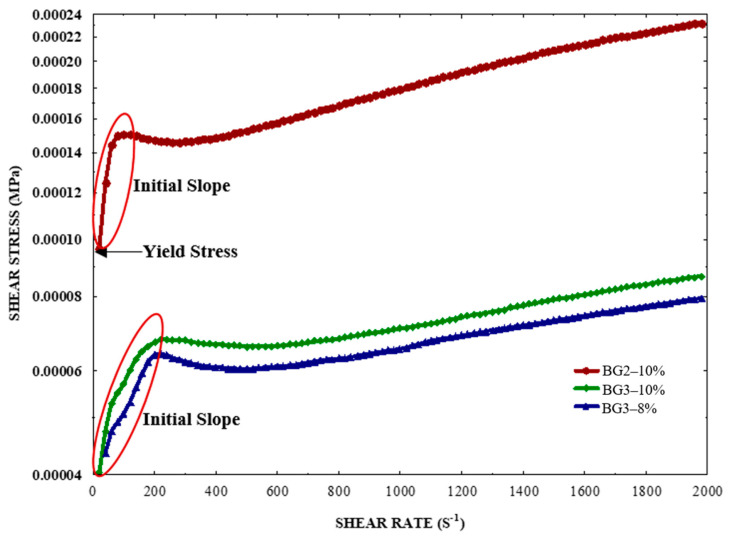
Rheological analysis of emulsion formulations: shear stress vs. shear rate. The red circle highlights the initial slope observed in the early stages of each emulsion’s behavior.

Rheological measurements (Figure 5) reveal that all three samples exhibit non-Newtonian behavior and shear-thinning (pseudoplastic). The W/O BG2–10% emulsion shows the highest yield stress, indicating that flow is initiated only after exceeding a specific force threshold. Below this threshold, the emulsion behaves like a solid, and the steep initial slope (m = 9 × 10^−7^) is attributed to the high oleogel content, which thickens the emulsion and improves its rigidity and flow resistance. This behavior is consistent with the Herschel–Bulkley model (τ = τγ + Kγ^n^), which describes fluids that exhibit yield strengths and shear-thinning characteristics. In contrast, the higher content of the EWP solution in BG3–10% and BG3–8% leads to a decrease in apparent viscosity (Table 2). O/W emulsions (BG3–10% and BG3–8%) exhibit a lower yield strength and more fluid-like behavior, leading to a less steep initial slope (m = 1.33 × 10^−7^ and 1.22 × 10^−7^, respectively). The yield strength and initial slope correlate with increasing apparent viscosity (Table 2), influenced by the H/O ratio and EWP concentration. Alves-Barroso et al. [17] similarly found that the viscosity of bigels could be modulated by adjusting the H/O ratio. 

#### 2.4.2. Ramp Temperature Test 

**Figure 6:**
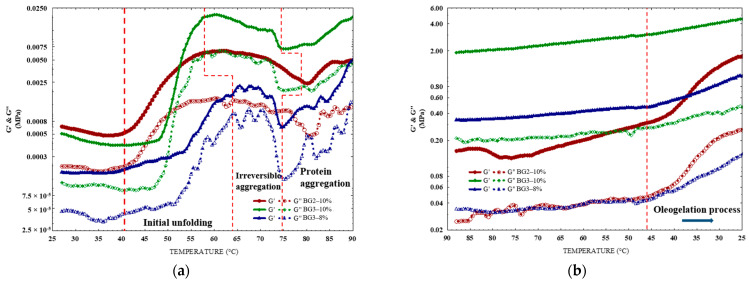
Rheological analysis of emulsion formulations: (**a**) heating and (**b**) cooling temperature sweeps.

A temperature sweep test (Figure 6a,b) was performed to determine the melting temperatures of the CW and the denaturation of EWP, which indicates the gelation process to obtain the BGs. The samples’ melting and denaturalization temperature patterns were consistent with their DSC temperature. Importantly, all the samples exhibited higher values of G′ (storage modulus) than those of G″ (loss modulus), showing a dominant elastic property (solid-like state) and indicating the strength of the developed BGs.

In the W/O BG2–10% emulsion, despite the continuous phase being CWO, G′ gradually increased from 39 °C, the onset of CW melting (Figure 6a, dotted red line). This increase is due to enhanced hydrophobic interactions between molten CW molecules and the hydrophobic regions of EWP. CW promoted the heat-induced unfolding of EW, causing exposure of the internal hydrophobic amino acids on the protein surface, and the protein structure continued to unfold [52]. This process likely involves an initial unfolding of EWP at low temperatures, exposing hydrophobic regions generally buried within the protein structure. This unfolding makes these regions more accessible for interactions. Previous studies have shown that egg white exhibits increased surface hydrophobicity between 30 and 40 °C [22], suggesting a more expanded protein structure. This expanded structure could further facilitate interactions with CW alkanes and potentially disrupt the tertiary protein structure, contributing to the significant increase in G′ observed when CWO is present.

The increase in G′ is much more pronounced in BG3–10% due to EWP’s higher protein content (as shown in Table 2), which increases protein–protein interactions in a more robust network structure. As a result, the storage modulus increases steeply during temperature sweeps due to the protein’s structural change, leading to a gel-like behavior due to the more cross-linked structure [53].

Conversely, BG3–8%, while maintaining the same oleogel content as BG3–10%, has a lower EWP concentration, likely resulting in weaker hydrophobic interactions between the fused CW crystals and protein molecules. Hydrophobic interactions are crucial in stabilizing the network structure and contributing to the G′ increase. Consequently, the increase in G′ in BG3–8% is less pronounced than in BG3–10%.

The maximum G′ observed between 60 and 65 °C likely represents the point of optimal hydrophobic interaction network formation between melted CW and EWP. As temperature increases, two competing processes occur: (1) complete CW melting, which may partially disrupt protein interactions; and (2) protein denaturation, leading to irreversible aggregation and formation of larger complexes, including oligomers [54]. While initial aggregation contributes to increased G′ at higher temperatures, these large aggregates can disrupt the gel network, leading to a decrease in G′ and a more brittle structure. Changes in protein conformation and the presence of water can also influence surface hydrophobicity [22], further impacting gel properties.

The increase in G′ above 80 °C is attributed to the denaturation of ovalbumin, the only egg white protein with free sulfhydryl groups, within the same temperature range observed by DSC analysis. Ovalbumin’s denaturation facilitates covalent bond formation with other egg white proteins via sulfhydryl groups [55], crucial in heat-induced gel formation [56,57]. This crosslinking, primarily through disulphide bridge formation, creates a three-dimensional network and strengthens the gel. The denaturation of ovalbumin is irreversible, resulting in the formation of an EWP-based hydrogel, as the entire CWO structure melts at this temperature.

During cooling, the chemical reorganization of intramolecular and intermolecular interactions, mainly hydrogen bonding and hydrophobic interactions, steadily increases the storage modulus (Figure 6b) [58]. Polar amino acids such as serine, threonine, tyrosine, asparagine, glutamine, and histidine facilitate hydrogen bonding through their hydroxyl, amide, and imidazole groups, which interact with water molecules or other polar side chains [59]. As temperature decreases, the reduced kinetic energy allows these polar side chains to interact more closely, stabilizing the protein structure and increasing the storage modulus. Additionally, hydrophobic interactions are crucial for stabilizing the protein network. To minimize exposure to the aqueous environment, non-polar amino acids such as leucine, isoleucine, valine, phenylalanine, and methionine tend to aggregate [59,60]. This aggregation enhances the structural integrity of the protein network and further increases the storage modulus by reducing the hydrophobic surface area exposed to water.

The increase in G′, observed at approximately 46 °C (Figure 6b, dotted red lines), coincides with the onset of CW crystallization and oleogel formation. This oleogelation process, occurring either internally within the hydrogel (BG3–10% and BG3–8%) or externally (BG2–10%), contributes to a more robust three-dimensional network structure and enhanced gel stability. This improved stability likely results from the synergistic interactions between oleogel and hydrogel, including van der Waals, hydrogen bonding, and London dispersion forces. Together, these interactions facilitate the formation of hybrid gels (bigels) with greater firmness.

#### 2.4.3. Amplitude Sweeps

**Figure 7:**
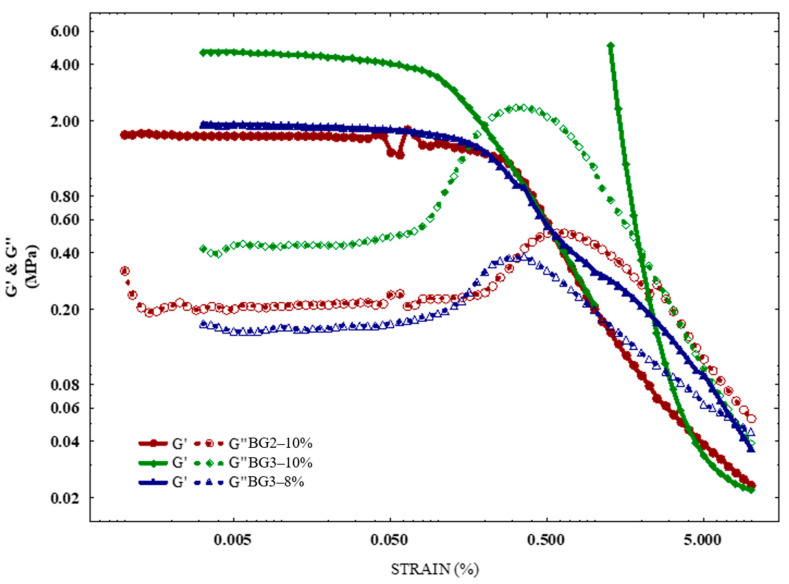
Amplitude sweeps, all with varying H/O ratios and EWP concentrations.

Figure 7 shows the amplitude sweeps of all BGs. The G′ value was higher than the G′′ value for all samples within the linear viscoelastic region (LVR), indicating that the bigels exhibited a gel-like solid structure. This region represents the strain amplitude range where the material behaves elastically and does not undergo structural breakdown under mechanical forces [17].

The LVR for BG2–10% (0.187 ± 0.020%) and BG3–8% (0.146 ± 0.020%) were statistically higher than that for BG3–10% (0.087 ± 0.004%). These results indicate that a higher oleogel ratio (BG2–10%) and lower protein concentration (BG3–8%) enhance system strength and stiffness. However, BG2–10% exhibited a prior decrease in G′ at approximately 0.05%, likely due to its higher oleogel content (60 *vol*/*vol*%), which suggests a more brittle structure and highlights the impact of CW oleogel on the bigel structure. This brittleness could be attributed to the denser and stronger protein network in BG3–10% due to a higher EWP concentration than BG2–10% and BG3–8%, making it more resistant to stretching. Stronger interactions between the filler materials (oleogel or hydrogel) might also contribute to the overall strength and deformation behavior of the bigel, leading to a more robust network and a higher strain value [61]. Above the LVR values, the storage modulus exhibited a pronounced decrease in the BG samples, indicating structural network breakdown at the crossover point (G′ = G′′). BGs with a higher oleogel component exhibit superior rheological properties, as the critical strain was higher, indicating their ability to withstand higher shear forces before transitioning to viscous flow. These results corroborate the textural analysis that identified the formation of strong network structures with a higher oleogelator (CW) content.

### 2.5. Microscopy Characterization

**Figure 8:**
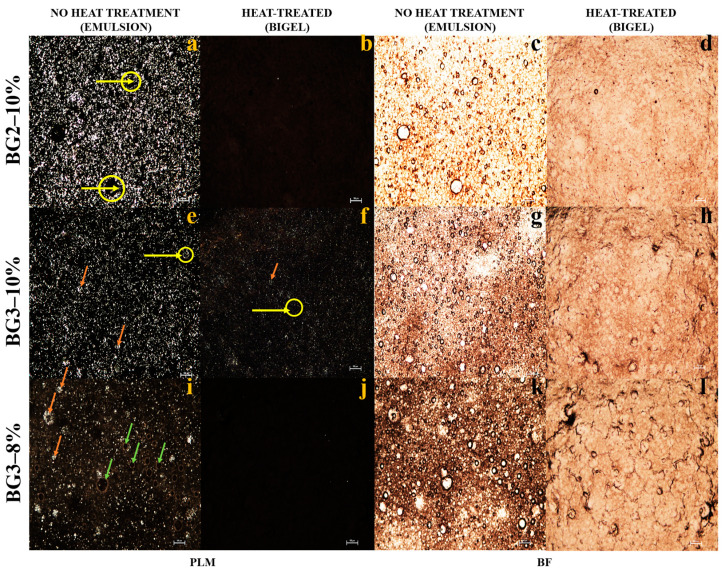
Polarized light microscopy (PLM, (**a**–**j**)) and brightfield microscopy (BF, (**c**–**l**)) images from left to right of emulsions (**left**) and bigel (**right**). Scale bar: 100 μm. Micrographs were obtained with a magnification of 4×. Yellow circles and arrows highlight the crystalized CW surrounding the dispersed phase. Orange arrows indicate the oleogel droplets, while green arrows point to the bubbles.

Confocal laser scanning microscopy (CLSM) and polarized light microscopy (PLM) were employed to evaluate the emulsion and oleogel stability and investigate their microstructure. As shown in Figure 8, the oleogel appears as a continuous phase with bright CW crystals due to birefringence, which is consistent with the findings of Toro-Vazquez et al. [8]. PLM reveals spherical oleogel droplets as bright CW aggregates within the hydrogel matrix (indicated by continuous orange arrows, Figure 8e,i). Brightfield microscopy (BF, Figure 8g,k) displays opacity in the continuous phase, attributed to protein aggregation and light-scattering structures influenced by protein concentration and interactions at the oil–water interface.

PLM images (Figure 8a,e,f) suggest that CW crystals are frequently located near the edges of the droplets (yellow circles and arrows), indicating their role as Pickering stabilizers [7,33]. EWP’s emulsifying and gelling properties demonstrate a synergistic effect between the oleogel and EWP, combining Pickering stabilization and protein network formation. The irregular shape of the aqueous droplets in the BG 3–10% and BG 2–8% bigels may result from collisions between CW crystals at the droplet interface [62].

**Figure 9:**
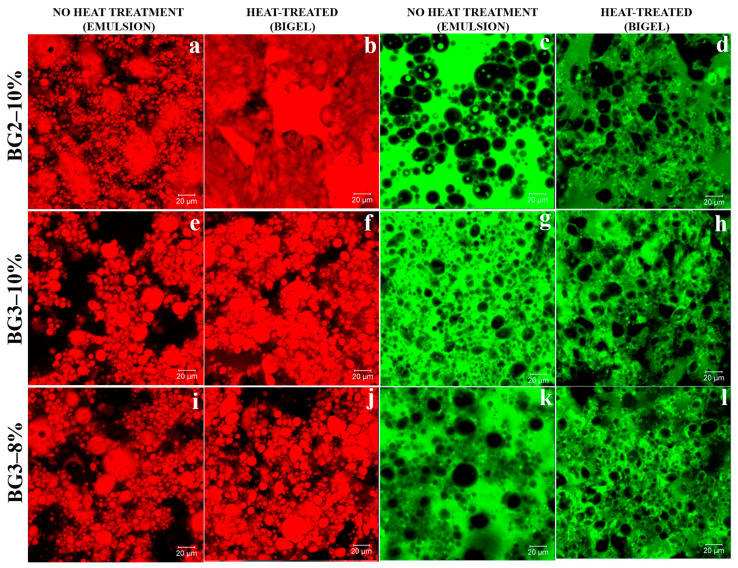
CLSM images of emulsions (left, (**a**–**j**)) and bigel (right, (**c**–**l**)). Oil staining by Nile red and EWP by FTIC from left to right. Scale bar: 20 μm. Micrographs were obtained with a magnification of 40×.

In the BG2–10% emulsion, the dispersed phase appeared in red-colored oleogel droplets (Figure 9a), while the aqueous phase was dark. This coloration likely results from protein adsorption at the oil–water interface, which influences the fluorescence distribution based on the polarity-dependent emission properties of the dye Nile red [63]. These results indicate that even at high oleogel contents, the proteins in the EW play a significant role at the oil–water interface.

Additionally, a dual network system was observed in the BG2–10% bigel: a green-stained protein network in the oil phase (Figure 9d), and a red-stained oleogel forming a semi-solid continuous phase (Figure 9b). This arrangement enhances the stability of the BG by providing structural reinforcement, improving resistance to mechanical stress. These findings suggest promising applications for creating stable, multifunctional materials in food, cosmetics, and pharmaceutical materials.

Confocal microscopy images of the BG2–10% bigel (Figure 9b) reveal that the oleogel (stained red) forms a dispersed phase characterized by heterogeneous coarse globules. In contrast, the EWP hydrogel (stained green) was the continuous phase in BG3–10% and BG3–8%. This observation confirms the successful formation of oleogel-in-hydrogel BGs.

During emulsification, EW proteins, particularly ovalbumin, rapidly adsorb to water–air and water–oil interfaces, reducing interfacial tension, trapping air, and facilitating film formation (Figure 8i, green arrow; Figure 9k) [64]. The absence of a distinct oleogel–hydrogel interface in the bigels indicates interactions between the CW crystals and the EW proteins, enabling both phases to function as active fillers [65].

At higher protein concentrations, the protein networks (Figure 9h) display increased tortuosity, which makes the BG3–10% bigel more susceptible to fracture at various stress points [66,67]. The protein structure consists of random spherical aggregates of ovotransferrin (in bigels at pH ≈ 7, near the isoelectric point of ovotransferrin) and branched linear aggregates of ovalbumin (pI ≈ 4.5, which is far from the pH of the bigels). This results in a complex network comprising both spherical and branched aggregates.

## 3. Conclusions

BG2–10% is identified as the optimal bigel formulation based on the rheological, microstructural, and stability analysis, as the higher oleogel content (60%) creates a structured oil phase that resists droplet movement and coalescence, as evidenced by lower TSI and consistent backscattering. Microscopy provides further evidence of a robust, continuous oleogel network. This structural integrity suggests that BG2–10% is best suited to applications where stability and strength are required, although further research is needed to assess its broader applicability.

The base emulsions for preparing oleogel and hydrogel exhibited high hydrophilicity due to the CW and EWP, forming a stable interfacial film and revealing hydrophilic regions. Consequently, the bigels maintain consistent strength and enhanced stability, aligning with the denser, more compact microstructure observed through microscopy. These characteristics were attributed to the EWP concentration and the hydrogel-to-oleogel ratio. A significant finding of this study was the determination of the melting points of CWO, which were influenced by the thermal conductivity of the hydrogel. This interaction positively impacts the final structure of the BG and its rheological properties. Furthermore, BGs with a higher oleogel ratio exhibited superior rheological characteristics, as indicated by higher critical strain, suggesting their ability to withstand greater shear forces before transitioning to viscous flow. Finally, microscopy studies further confirmed the formation of oleogel-in-hydrogel bigels, highlighting CW crystals acting as Pickering stabilizers, while EWP forms a continuous hydrogel network. These results demonstrate the potential of BG hybrid systems as possible components to create innovative foods with desirable nutritional, health, quality, textural, and sensory properties. These attributes can be tuned by adjusting the EWP concentration and the hydrogel-to-oleogel ratio.

## 4. Materials and Methods

### 4.1. Materials

Hen egg whites (Huevo San Juan^®^) were obtained from local suppliers, and distilled water was utilized for hydrogels preparation. Oleogels were formulated using commercial soybean oil (Nutrioli^®^, Ragasa Industrias, S.A. de C.V., Monterrey, Nuevo León, Mexico), while candelilla wax was supplied by Bliss Nature (Mexico City, Mexico). Bovine serum albumin (BSA) was purchased from Sigma-Aldrich (St. Louis, MO, USA). All other chemical reagents were of analytical grade.

### 4.2. Protein Determination

The protein content was determined using a modified Biuret method based on the procedure outlined by Gornall et al. [68]. Bovine serum albumin (BSA, Sigma-Aldrich) was dissolved in 0.1 M NaOH and filtered through Whatman^TM^ filter paper N° 3 to create a calibration curve. Egg whites were manually separated, diluted 1:50 in 0.1 M NaOH, homogenized for 30 min, and filtered through Whatman^TM^ filter paper N° 3 before analysis.

### 4.3. Bigel Elaboration

The bigels were produced in three stages, as follows:

Aqueous phase (hydrogel): Egg whites (EWs) were homogenized in a low-speed beaker for 30 min to minimize foaming. The mixture was then allowed to stand for 1 h before further dilutions at six concentrations: 5, 6, 7, 8, 9, and 10% (*w*/*v*). Each dilution was vortex-mixed for 1 min and incubated at 8 °C for 24 h prior to use.

Oily phase (oleogel): Candelilla wax oleogel (CWO) was prepared according to the method described by Cisneros and Totosaus [69]. CW was dispersed in soybean oil at a concentration of 7.5% (*w*/*w*), CWO7.5. CWO was melted at 90 °C in an oven for 25 min, mixed for 1 min, and then reheated at 90 °C for 10 min. The oleogel was stored at room temperature for 24 h before further analysis.

**Figure 10:**
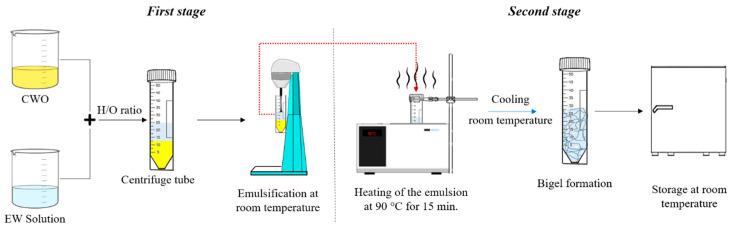
Formation of the bigel involves two stages: first, the emulsification process, followed by the emulsion gelling process.

Bigels: These were produced by combining the aqueous and oily phases (Figure 10). Centrifuge tubes were used for their preparation. The names and compositions of the samples are shown in Table 1. The procedure comprised two stages: emulsion formation and gel formation. In the first stage, egg white emulsions (EW: 5, 6, 7, 8, 9, and 10%) were mixed with oleogel (CWO7.5) and sheared at low speed for 2 min using a commercial homogenizer (Model RCA-219). In the second stage, the emulsions were heated in a water bath at 90 °C for 15 min and then cooled to room temperature to facilitate the formation of egg white-based bigels. These bigels were then stored for 24 h before further analysis.

### 4.4. Bigels Compression Tests

The compression test was conducted using the methodology outlined by Cisneros and Totosaus [69] with minor modifications. Cylindrical samples of bigels, measuring 15 mm in length and 23 mm in diameter, were carefully removed from the tubes for analysis. These samples underwent axial compression at a strain rate of 20% using a TA4/1000 probe in a texture analyzer (Brookfield CT3 50K, Engineering Laboratories, Middleboro, NJ, USA) at a spindle speed of 1 mm/s. The maximum compressive force was determined from the force–strain curves generated during the testing. The tests were carried out with three replicates and three duplicates.

### 4.5. Contact Angle Measurements

The hydrophobicity and lipophilicity of the emulsions were assessed by measuring contact angles without heat treatment. Emulsion samples were deposited uniformly on slides at room temperature. Static contact angles were determined by placing water or soybean oil drops on the sample surfaces using a CAM-Plus Contact Angle Meter (Cole-Parmer^®^, Vernon Hills, IL, USA). The subsequent analysis was conducted using ImageJ software. The tests were carried out with three replicates and three duplicates.

### 4.6. Emulsions Stability

The stability of the emulsions was evaluated using Turbiscan^®^ Lab equipment (Formulation Smart Scientific Analysis, Lille, France) during the initial stage of bigel preparation without heat treatment. First, 20 mL was drawn using a 60 mL syringe fitted with a 5 mm × 3 mm (F16) × 1000 mm hose to minimize spillage. Stability scans were conducted for five days. Finally, the Turbiscan Stability Index (TSI) was calculated to capture all instability phenomena. The tests were conducted in triplicate.

### 4.7. Apparent Viscosity Measurements of Emulsions

The apparent viscosity of the emulsions was assessed using a Brookfield rheometer (model RST CC, Brookfield Engineering Labs Inc., Middelboro, MA, USA) with concentric cylinder geometry. Measurements were conducted at room temperature to ensure accuracy. The apparent viscosity was determined from the flow curve graph of shear rate versus shear stress, where the slope of the plotted curve was calculated. All tests were performed in triplicate.

### 4.8. Differential Scanning Calorimetry (DSC)

Differential scanning calorimetry (DSC) was performed to analyze the thermal transition profiles. A calorimeter from TA Discovery Instruments equipped with a cooling unit (TA Instruments, New Castle, DE, USA) was used. For this analysis, an empty hermetically sealed aluminum pan served as reference. Emulsion samples (14–16 mg) were subjected to a heating protocol. The first stage of bigel formulation without heat treatment was loaded into the sample pan and held isothermally at 25 °C for 2 min. Then, the samples were heated from 25 °C to 90 °C at a rate of 10 °C/min, followed by a 15 min isothermal hold at 90 °C and cooling back to 25 °C at 10 °C/min. Thermal parameters and thermograms were analyzed using TRIOS 5.5.1 software (TA Instruments), with all measurements performed in triplicate. The tests were conducted in triplicate.

### 4.9. Rheological Behavior

The rheological properties of the emulsions were analyzed through heating ramps, isothermal treatments, cooling processes, and deformation sweeps. Measurements were conducted using a Discovery Hybrid HR3 rheometer (TA Instruments Ltd., New Castle, DE, USA) with a 40 mm diameter parallel plate and 500 μm gap. Emulsions were heated from 25 °C to 90 °C at 10 °C/min within the linear viscoelastic region at 0.1 Hz, evaluating bigel formation via changes in storage modulus (G′) and loss modulus (G′′). A 15 min isothermal treatment was applied at 90 °C, followed by cooling to 25 °C, and another 15 min isothermal treatment at 25 °C, with a frequency of 1.0 Hz, covering a logarithmic strain range from 0.001% to 100%. Flow tests were conducted on emulsions using the same rheometer setup, with a shear rate sweep from 0 to 2000 s⁻¹ performed over 1 min after a 120 s equilibration at 25 °C. The tests were carried out with three replicates and three duplicates.

### 4.10. Confocal Laser Scanning Microscopy

Confocal laser scanning microscopy (CLSM) was employed to analyze emulsions and bigels using a CLSM LSM700 microscope (Carl Zeiss, Oberkochen, Germany). Protein in the emulsions was stained by mixing 0.25 mL of 0.01% (*w*/*v*) fluorescein-5-isothiocyanate (FITC) with EW dilutions and homogenizing with CWO7.5. For oleogel staining, Nile red (0.01% *w*/*w*) was mixed with oil, shaken for 1 h, and filtered (WhatmanTM N° 3). The stained oil was then incorporated into CWO7.5 following the bigel preparation procedure. All the samples were observed at 25 °C using a 40× objective.

### 4.11. Polarized Light Microscopy

The microstructure of the emulsions was examined using a Nikon Eclipse 80i polarized light microscope (Nikon Corp., Tokyo, Japan). Images were captured at 4×, 10×, and 20× magnifications using an MTI DC330 CCD digital camera (Oxford Ins., Abingdon, UK). The sample temperature was controlled with a heating module connected to a PE94 temperature control station (Linkam Scientific Instruments, Ltd., Surrey, England). The samples were deposited on slides equilibrated to 25 °C, heated from 25 °C to 90 °C at 10 °C/min, held at 90 °C for 15 min, and then cooled back to 25 °C for analysis.

### 4.12. Statistical Analysis

Experiments were conducted in randomized order to minimize variability. Tukey’s HSD post hoc test was employed for group mean comparisons at *p* < 0.05 significance. Statistical analysis was performed using Statistica 10.0 (StatSoft, Tulsa, OK, USA), while OriginPro 2019 (OriginLab Corp., Northampton, MA, USA) was used for baseline generation and data smoothing.

## Figures and Tables

**Table 1:** Formulation and composition of bigels with varying EWP concentrations and CWO fractions.

Sample	EWP Concentration (%*w*/*v*)	Hydrogel (H)/Oleogel (O) Ratio	Final EW Concentration (%*w*/*v*)	Final CW Concentration (%*w*/*v*)
BG1–10%	10	20/80	2.18	6
BG2–10%	10	40/60	4.36	4.5
BG3–10%	10	60/40	6.55	3
BG4–10%	10	80/20	8.72	1.5
BG1–9%	9	20/80	1.96	6
BG2–9%	9	40/60	3.92	4.5
BG3–9%	9	60/40	5.88	3
BG4–9%	9	80/20	7.84	1.5
BG1–8%	8	20/80	1.7	6
BG2–8%	8	40/60	3.41	4.5
BG3–8%	8	60/40	5.1	3
BG4–8%	8	80/20	6.82	1.5
BG1–7%	7	20/80	1.51	6
BG2–7%	7	40/60	3.02	4.5
BG3–7%	7	60/40	4.53	3
BG4–7%	7	80/20	6.04	1.5
BG1–6%	6	20/80	1.27	6
BG2–6%	6	40/60	2.56	4.5
BG3–6%	6	60/40	2.83	3
BG4–6%	6	80/20	5.11	1.5
BG1–5%	5	20/80	1.03	6
BG2–5%	5	40/60	2.07	4.5
BG3–5%	5	60/40	3.1	3
BG4–5%	5	80/20	4.14	1.5

**Table 2:** Comparison of contact angle and apparent viscosity at varying H/O ratios and EWP concentrations.

Bigel	Contact Angle Using Oil Drop	Contact Angle Using Water Drop	Apparent Viscosity
	(°)	(°)	(P × s)
BG2–10%	38.93 ± 3.35 ^a^	<1 ^a^	0.1776 ± 0.010 ^a^
BG3–10%	33.39 ± 4.86 ^ab^	<1 ^a^	0.0596 ± 0.001 ^b^
BG3–8%	30.62 ± 1.67 ^b^	<1 ^a^	0.0460 ± 0.001 ^b^

*p*-values for the ANOVA of the effect of H/O ratios and EWP concentrations. Means within each column followed by the same letter are not significantly different (α = 0.05).

## Data Availability

The data presented in this study are available upon request from the corresponding author.
